# Geo-temporal distribution of 1,688 Chinese healthcare workers infected with COVID-19 in severe conditions—A secondary data analysis

**DOI:** 10.1371/journal.pone.0233255

**Published:** 2020-05-14

**Authors:** Wayne Gao, Mattia Sanna, Min Kuang Tsai, Chi Pang Wen

**Affiliations:** 1 Master’s Program in Global Health and Development, Taipei Medical University, Taipei, Taiwan; 2 Institute of Population Health Science, National Health Research Institutes, Zhunan, Taiwan; 3 China Medical University Hospitals, Taichung, Taiwan; Faculty of Science, Ain Shams University (ASU), EGYPT

## Abstract

**Introduction:**

The COVID-19 outbreak is posing an unprecedented challenge to healthcare workers. This study analyzes the geo-temporal effects on disease severity for the 1,688 Chinese healthcare workers infected with COVID-19.

**Methods:**

Using the descriptive results recently reported by the Chinese CDC, we compare the percentage of infected healthcare workers in severe conditions over time and across three areas in China, and the fatality rate of infected healthcare workers with all the infected individuals in China aged 22–59 years.

**Results:**

Among the infected Chinese healthcare workers whose symptoms onset appeared during the same ten-day period, the percentage of those in severe conditions decreased significantly from 19.7% (Jan 11–20) to 14.4% (Jan 21–31) to 8.7% (Feb 1–11). Across the country, there was also a significant difference in the disease severity, with Wuhan being the most severe (17.3%), followed by Hubei Province (10.2%), and the rest of China (6.6%). The case fatality rate for the 1,688 infected Chinese healthcare workers was significantly lower than that for the 29,798 infected patients aged 20–59 years—0.3% (5/1,688) vs. 0.65% (193/29,798), respectively.

**Conclusion:**

The disease severity among infected healthcare workers improved considerably over a short period of time in China. The more severe conditions in Wuhan compared to the rest of the country may be attributable to the draconian lockdown. The clinical outcomes of infected Chinese healthcare workers may represent a more accurate estimation of the severity of COVID-19 for those who have access to quality healthcare.

## Introduction

The first cluster of novel coronavirus disease 2019 (COVID-19) originated in Wuhan, China on December 31, 2019[1]. By April 23, 2020, 2.64 million cases of COVID-19, including 184,268 deaths, had been confirmed, beginning mainly in China and now in more than 185 countries and areas[2]. Severity and transmissibility of the COVID-19 have been the most important issues researchers and public health authorities have been urgently trying to tackle since the start of the outbreak. The first studies reporting single-center case series of hospitalized patients of COVID-19 showed high rates of admission to intensive care (32%), high mortality (15%)[3], and case fatality rates (CFR) of 4.3% (6/138)[4] and 11% (11/99)[5] in Wuhan, while lower figures were reported outside the city[6]. However, the high CFRs observed in Wuhan are likely to be overestimated, as the cited studies have mainly considered patients with severe symptoms who were hospitalized, excluding mild and asymptomatic patients who are less likely to be admitted to the hospital[7]. The most recent estimation of an overall 2.3% CFR reported by China CDC[8,9] is thus tentative, and more specific figures will remain undetermined until a later point.

Frontline healthcare workers are significantly more likely than general public to come into contact with infected individuals. Knowing the clinical outcomes of healthcare workers who have been infected may provide critical information on both risk and disease severity, especially when proper healthcare services are available. Here, we examine if there was a difference in the severity of COVID-19 among infected healthcare workers in China both geographically, across three different locations, and temporally, over three ten-day periods, based on the dates of symptoms onset during the initial period of the COVID-19 epidemic.

## Methods

We use the descriptive results from “The epidemiological characteristics of an outbreak of 2019 novel coronavirus disease”, published by the Chinese CDC[8], which is, by far, the most comprehensive epidemiological investigation of COVID-19 in China. This report covers all the 72,314 COVID-19 cases and the 1,688 laboratory-confirmed cases among healthcare workers as of February 11. A similar version in English was also published in the Journal of American Medical Association (JAMA)[9], including clinical outcomes, classified by severity (mild symptoms vs. severe/critical conditions) and by time into three 10-day periods based on the onset of symptoms reported by the patients. Using chi-squared test and Fisher’s exact test, we analyze the changes in percentage of infected healthcare workers in severe conditions over the same three periods (1/11-1/20, 1/21-1/31, and 2/1-2/11) and across the same three regions (Wuhan City, Hubei Province excluding Wuhan, China excluding Hubei). We also compare the CFR for all infected patients aged 20–59 and for healthcare workers.

## Results

**Fig 1:**
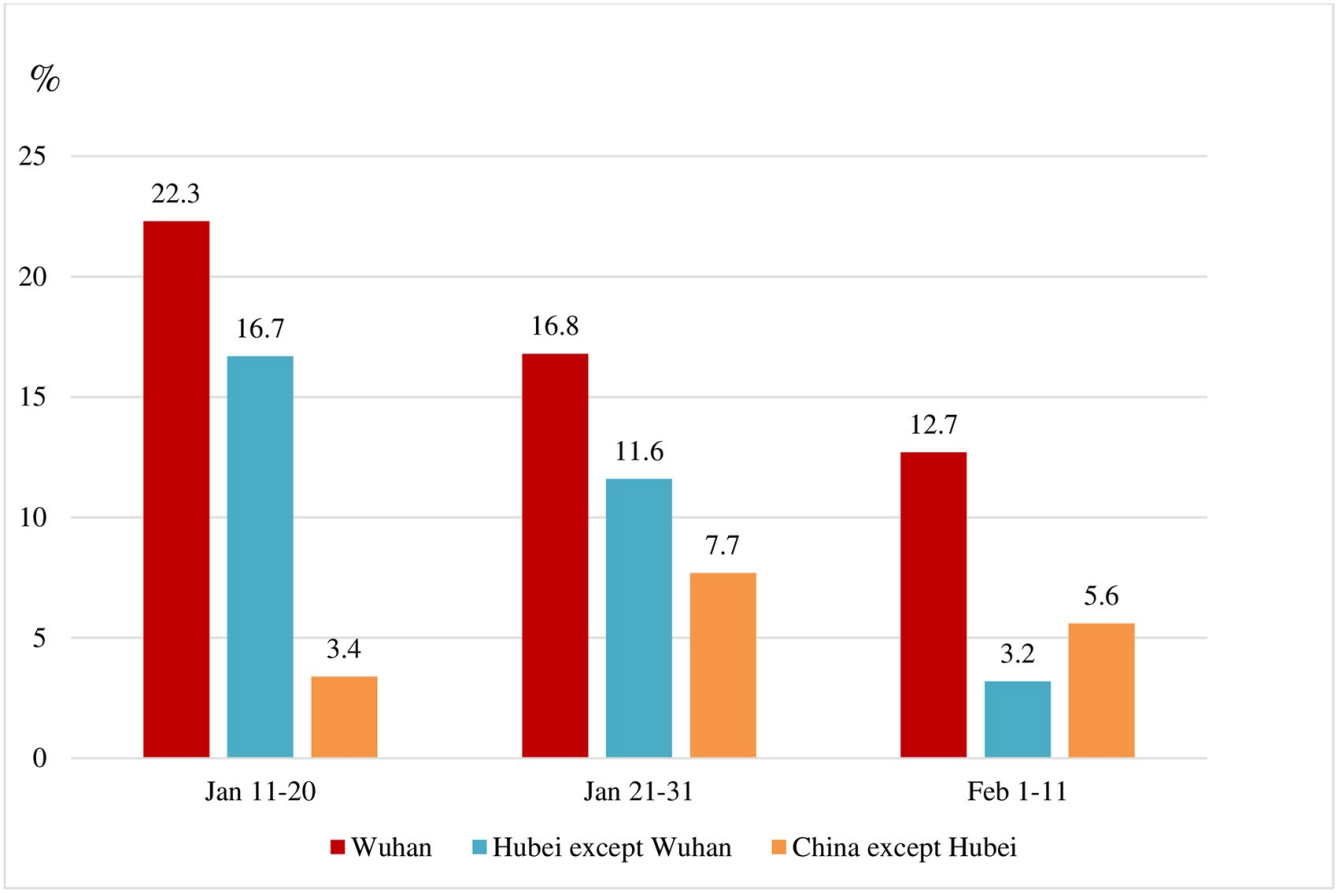
Percentage of healthcare infected workers in severe conditions by dates of symptoms onset and regions in China.

The CFR for the 1,688 infected Chinese medical workers was significantly lower than the CFR for the 29,798 infected patients aged 20–59 years—0.3% (5/1,688) vs. 0.65% (193/29,798), respectively. Aggregating the data of the three regions, the percentage of healthcare workers in severe conditions decreased significantly, from 19.7% (Jan 11–20) to 14.4% (Jan 21–31) to 8.7% (Feb 1–11). The same pattern can be observed in Wuhan and in Hubei province, but not in the rest of China, as shown in Fig 1.

Considering only patients whose symptom onset appeared during the same 10-day period, the percentage of those in severe conditions exhibits a distinctive geographic distribution centered around the lockdown epicenter of the outbreak—Wuhan being the most severe (17.3%), followed by Hubei Province excluding Wuhan (10.2%), and by of China excluding Hubei (6.6%) (Table 1). Of the 149 Chinese health workers who were infected with COVID-19 outside of Wuhan City after February 1, only 4% were in severe condition (6/149) and there were no deaths.

**Table 1:** Statistical analysis of COVID-19 cases among healthcare workers in China.

	Wuhan			Hubei except Wuhan			China except Hubei			Fisher’s exact test
Dates	Confirmed cases	Severe cases	(%)	Confirmed cases	Severe cases	(%)	Confirmed cases	Severe cases	(%)	
**Jan 11–20**	233	52	(22.3)	48	8	(16.7)	29	1	(3.5)	0.001
**Jan 21–31**	656	110	(16.8)	250	29	(11.6)	130	10	(7.7)	0.01
**Feb 1–11**	173	22	(12.7)	95	3	(3.2)	54	3	(5.6)	0.001
**Chi-square test**			0.03			0.0004			0.07	
Total	1062	184	(17.3)	393	40	(10.2)	213	14	(6.6)	<0.0001

**Fig 2:**
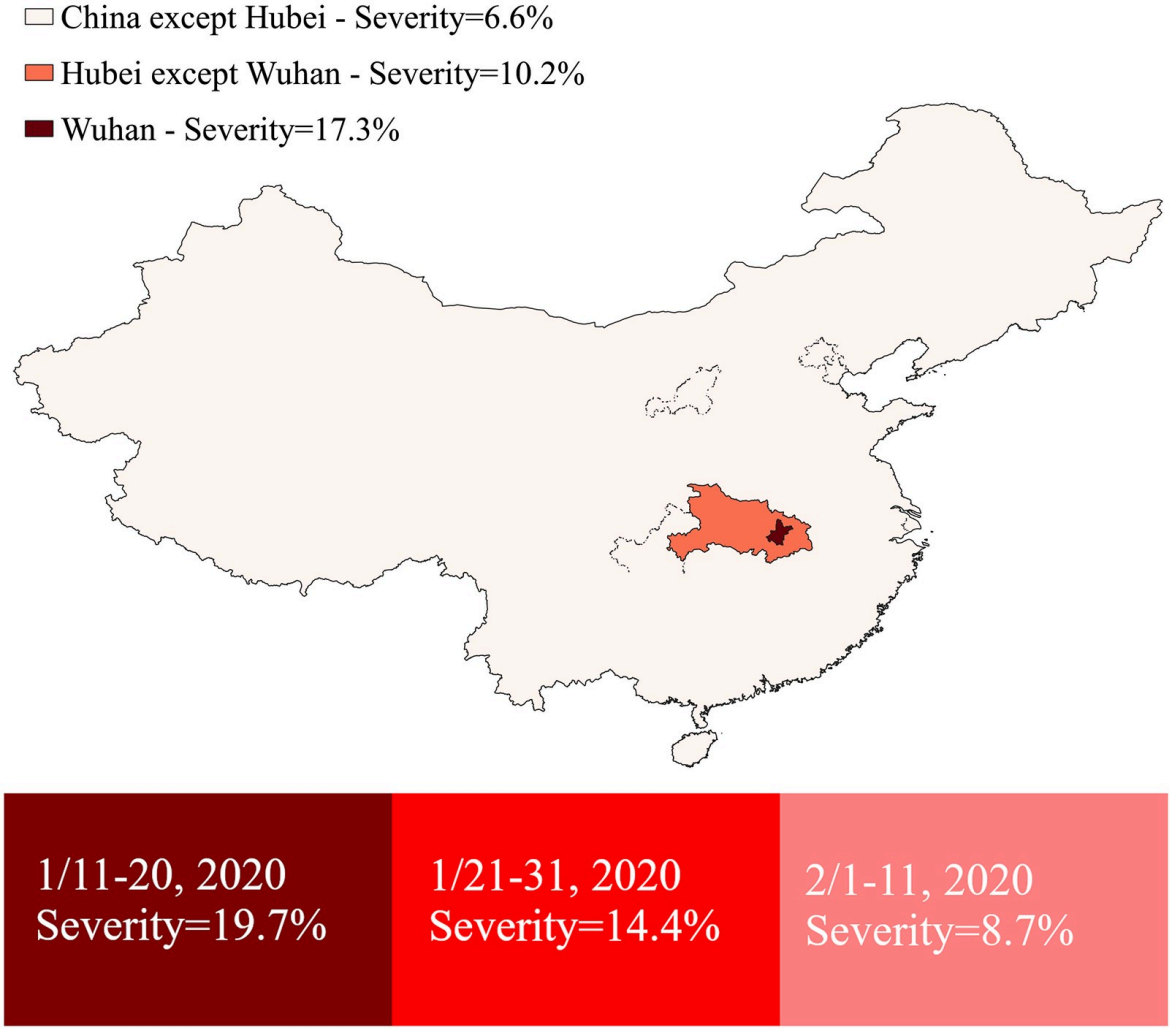
Percentage of COVID-19 infected healthcare workers in severe conditions in different geographic locations and over three ten-day periods, based on symptom onset dates. This map was created by authors with QGIS 3.12, using shapefiles available from The Humanitarian Data Exchange (https://data.humdata.org/).

We use a simple radiative map of the disease severity from Wuhan, Hubei, and the rest of China, as well as a chart to illustrate more intuitively the evolution over time and over space (Fig 2).

## Discussion

Preventing nosocomial transmission is a top priority during an epidemic. The consistent higher percentage of COVID-19 infected healthcare workers in severe conditions in Wuhan may be related to the draconian city lockdown and the established of massive quarantines camps, since viral factors such as mutation or adaptation are unlikely to be responsible for the difference observed for the three 10-day periods (as they occur over longer periods) and across the three locations taken into account. The transmission control was improper and co-infections were common during the initial period of the outbreak in Wuhan. One recent study found a correlation between the higher CFR in Wuhan with the higher healthcare burden compared with other provinces in China, assuming average levels of health care are similar throughout China[10]. However, according to National Health Commission of the People’s Republic of China, As of March 31, 42,600 health care workers were sent to Hubei Province to care for patients with Covid-19 and surprisingly, none of them have been infected with severe acute respiratory syndrome coronavirus 2 (SARS-CoV-2)[11]

It is unthinkable that sickening citizens, infected or not, and family members were confined in a city lockdown with an overwhelmed medical system and that they were forbidden from going to other provinces to seek for better care to save their lives. This also inevitably created logistical as well as psychological impact on healthcare workers operating in those harsh environments, described in a call for international assistance by Chinese medical workers, as “nurses’ mouths are covered in blisters and some nurses have fainted due to hypoglycaemia and hypoxia”[12]. Sixty-three percent of the infected healthcare workers have been in Wuhan, including the 5 who died as a result of the infection[9]. However, the 0.3% CFR for the 1,688 medical workers (who were mainly aged less than 60 years as indicated in the report [8]), may represent a proper estimation of the severity of COVID-19 in this age group in countries with universal access to quality medical services.

Our study has a few limitations. First, relevant risk factors such as age, gender, and pre-existing conditions might have been different among healthcare workers in different geographic locations, which might have affected their disease outcomes. Second, there may be infected healthcare workers who still have not completed their natural progression of the disease with definite outcomes. However, as of February 28, no additional deaths of healthcare workers were reported. Third, our data analysis is based on a governmental investigation of the outbreak, thus the validity of the descriptive results in the report inevitably determines the accuracy of our secondary analysis. For instance, the Chinese government recently revised the COVID-19 death toll in Wuhan by 50% more after three months. This update makes the COVID-19 case fatality rate increased from 3% to 6.6% in Hubei as of April 23, 2020. However, this new update is in line with our findings that an unusual COVID-19 burden among infected individuals, including healthcare workers, has occurred in Wuhan. Such discrepancy needs further independent investigation with access to individual, demographic and epidemiological data.

The spectrum of clinical severity of a novel communicable disease is critical in knowing the potential impact of an ongoing epidemic. Our finding that the spectrum of COVID-19 severity decreases eccentrically from the epicenter as well as over short periods of time, will help to avoid drastic, costly, and fear-driven measures that impede not only daily activities, but also a proper containment of the epidemic.

## Supporting information

S1 Data (XLSX)
